# Impact of long-term viral suppression in CD4+ recovery of HIV-children on Highly Active Antiretroviral Therapy

**DOI:** 10.1186/1471-2334-6-10

**Published:** 2006-01-24

**Authors:** Salvador Resino, Rosa Resino, Juan A Leon, José M Bellon, Pablo Martin-Fontelos, Jose T Ramos, Dolores Gurbindo-Gutierrez, Maria I de Jose, Luis Ciria, Maria A Muñoz-Fernandez

**Affiliations:** 1 Laboratorio de Inmuno-Biología Molecular, Hospital General Universitario "Gregorio Marañón", Madrid. Spain; 2 Servicio de Pediatría-Infecciosas; Hospital Universitario "Virgen de Rocío", Sevilla. Spain; 3 Servicio de Pediatría-Infecciosas; Hospital Universitario "Carlos III", Madrid. Spain; 4 Servicio de Inmuno-Pediatría; Hospital Universitario "12 de Octubre", Madrid. Spain; 5 Servicio de Inmuno-Pediatría; Hospital General Universitario "Gregorio Marañón", Madrid. Spain; 6 Servicio de Inmuno-Pediatría; Hospital Universitario "La Paz", Madrid. Spain; 7 Servicio de Pediatría-Infecciosas; Hospital Universitario "Niño Jesús". Madrid, Spain

## Abstract

**Background:**

The effects of HAART may differ between children and adults because children have a developing immune system, and the long-term immunological outcome in HIV-infected children on HAART is not well-known. A major aim of our study was to determine CD4+ evolution associated with long-term VL control during 4 years of observation on HAART.

**Methods:**

We carried out a retrospective study on a cohort of 160 vertically HIV-infected children. It was carried out from 1996 to 2004 in six large Spanish pediatric referral hospitals. We compared 33 children who had long-term VL suppression (VL ≤400 copies/ml) in the first 12 months of follow-up and maintained that level throughout follow-up (Responders-group), and 127 children with persistently detectable VL in spite of ART switches (Non-Responders-group).

**Results:**

We observed a quick initial and significant increase in CD4^+ ^counts from the baseline to 12 months on HAART in both groups (p < 0.01). The *Non-Responders group *sustained CD4+ increases and most of these children maintained high CD4^+ ^level counts (≥25%). The *Non-Responders group *reached a plateau between 26% and 27% CD4^+ ^at the first 12 months of follow-up that remained stable during the following 3 years. However, the *Responders group *reached a plateau between 30% and 32% CD4^+ ^at 24, 36 and 48 months of follow-up. We found that the *Responders group *had higher CD4^+ ^count values and higher percentages of children with CD4^+ ^≥25% than the *Non-Responders group *(p < 0.05) after month 12.

**Conclusion:**

Long-term VL suppression in turn induces large beneficial effects in immunological responses. However, it is not indispensable to recover CD4^+ ^levels.

## Background

The efficacy of highly active antiretroviral therapy (HAART) is shown by the fact that many patients achieve suppression of viral load (VL) below the limits of detection (uVL) along with an increase of CD4^+ ^T-lymphocytes (CD4^+^) [1,2], resulting in a good clinical outcome [3,4]. Immediate suppression of VL is often achieved with HAART, but long-term suppression of VL is not always feasible [1]. Children receiving treatment usually have higher VL and lower virologic response rates than adults [1,5]. Moreover, the effects of HAART may differ between children and adults because children have a developing immune system, but the long-term immunological outcome in HIV-infected children on HAART is not well-known [6]. A major aim of our study was to determine CD4+ evolution associated with long-term VL control.

## Methods

### Population and study design

A retrospective study on a cohort of 160 vertically HIV-infected children has been carried out from 1996 to 2004 in six large Spanish pediatric referral hospitals. The inclusion criteria of HIV-1-infected children were the following: a) starting HAART with protease inhibitor (PI) or non-nucleoside analogue HIV-1 reverse transcriptase inhibitor (NNRTI); b) 4 years of follow-up after starting HAART; c) VL >5,000 copies/ml at entry to the study, d) older than 6 months of age at entry. From an initial cohort of 200 vertically HIV-1-infected children with at least 4 years on HAART, 40 children were excluded because they had VL <5,000 copies/ml (30 children), no data regarding VL (1 child), or they were less than 6 months old (9 children). Therefore, 160 out 200 HIV-infected children fulfilled the criteria of inclusion and were enrolled in the present retrospective study.

This study was approved by the Ethical Committees of all hospitals involved. Clinical classification was based on the 1994 revised guidelines of the Centers for Disease Control (CDC). The children were monitored at least every 3 months with repeated interviews, physical examinations, and blood sample collection. There was not an uniform approach regarding antiretroviral treatment. Instead, each pediatrician administered the appropriate antiretroviral therapy (ART) regimen and changed the drugs according to his/her interpretation of the child's data and following international guidelines. The adherence of antiretroviral drugs was measured by each pediatrician by examination of the dose taken by each child and through interviews with his parents or tutors.

### Response to long-term HAART

A completed long-term virological response to HAART was defined when HIV-child achieved undetectable VL (≤400 copies/ml) in the first 12 months of therapy and maintained that level during at least 12 months. Thus, HIV-infected children were divided into two groups according to responses to long-term HAART:

#### a) Responders group

33 HIV-infected children with a full long-term virological response during at least 12 months. Detectable VL during a 12 months period, even if a small "blip", disqualified children from being included in the *Responders group*. Moreover, 29 of 33 had full long-term virological response during at least 24 months, and 25 of 33 had full long-term virological response during at least 36 months.

#### b) Non-Responders group

127 HIV-infected children with persistently VL despite HAART (VL ≥400 copies/ml during follow-up). These HIV-infected children had prolonged virological failure in spite of ART switches.

### HIV-1 infection laboratory markers

T-lymphocyte subsets in peripheral blood were quantified by flow cytometry (FACScan, Becton-Dickinson Immunocytometry Systems, San Jose, CA, USA). VL was measured in 200 μl plasma samples using a quantitative assay (Amplicor monitor, Roche Diagnostic Systems, Brandenburg, NJ, USA).

### Statistical analysis

We analyzed the CD4^+ ^at 0, 12, 24, 36, and 48 months follow-up of children on HAART grouped according to response to long-term virological suppression with a General Lineal Model (GLM) Univariate (regression analysis) adjusted by baseline characteristics (age, sex, ART-naïve, CD4+, and VL). In addition, we analyzed immunological response to HAART in HIV-infected children (CD4^+ ^>25% at 12, 24, 36, 48 months follow-up) by logistic regression.

## Results

At baseline (before starting HAART), the two groups had similar age, percentage in clinical category C, levels of CD4^+ ^and CD8^+ ^T-cells, and VL (Table 1). However, the Non-Responders group had a higher percentage of the pre-treatment with ART.

**Table 1:** Characteristics of clinical, immunological, and virological parameters of vertically HIV-1-infected children.

	***Responders***	***Non-Responders***
**N. of HIV-infected children**	33	127
**Age (years) **^(a)^	5.9 (1.2; 15.7)	5.7 (0.5; 14.9)
**Male **^(b)^	17 (51.5%)	58 (45.6%)
**AIDS diagnosis (CDC) **^(b)^	13 (39.3%)	58 (45.6%)
**Current immunological category **^(b)^		
> 25% CD4^+^	8 (24.2%)	40 (31.4%)
15% – 25% CD4^+^	9 (27.2%)	31 (24.4%)
< 15% CD4^+^	16 (48.4%)	52 (40.9%)
**Immunological parameters **^(a)^		
% CD4^+^	12 (0.1; 35)	18 (0.1; 52)
CD4^+^/μL	333 (4; 1207)	440 (1; 2620)
% CD8^+^	49 (22.1; 82)	47 (13; 74.6)
CD8^+^/μL	927 (238; 3763)	1006 (17; 6466)
**Virological characteristics**		
log_10 _VL (copies/mL) ^(a)^	4.8 (3.7;6.4)	4.8 (3.7; 6.5)
VL ≤10,000 copies/mL ^(b)^	6 (18.1%)	8 (6.2%)
VL 10,000 to 30,000 copies/mL ^(b)^	7 (21.2%)	28 (22.1%)
VL 30,000 to 100,000 copies/mL ^(b)^	11 (33.3%)	35 (27.5%)
VL >100,000 copies/mL ^(b)^	9 (27.2%)	56 (44.1%)
**ART prior to HAART**		
**ART-naïve HIV-infected children **^(b)^	13 (39.4%)	17 (13.4%)*
**HIV-infected children with previous ART **^(b)^		
Monotherapy	15 (45.4%)	71 (55.9%)
Combined Therapy	18 (54.5%)	99 (77.9%)*
Monotherapy + Combined Therapy	20 (60.6%)	110 (86.6%)*
**Time (months) with previous ART ^(a)^**	25.9 (0; 75.8)	22.4 (0; 114.1)
**Previous ART-protocol switches ^(a)^**	2 (0; 4)	2 (0; 5)

Values are expressed as: a) median (min; max), and b) absolute (percentage). VL: viral load; CDC = Center for Disease Control. VL: viral load. Differences between groups (*: p < 0.01).

During follow-up, one child progressed to AIDS and one child died. Moreover, the *Non-Responders group *presented a significantly higher number of ART switches, higher number of different drugs used in ART than children of the *Responders group *(p ≤ 0.05) (Table 1).

Long-term viral load suppression was achieved in only about 20% of followed children. This low figure is mostly related to poor adherence by the non-responders group (48% vs 91% in responders) (Table 1). There were not significant differences in adherence according to hospital and region involved.

**Figure 1:**
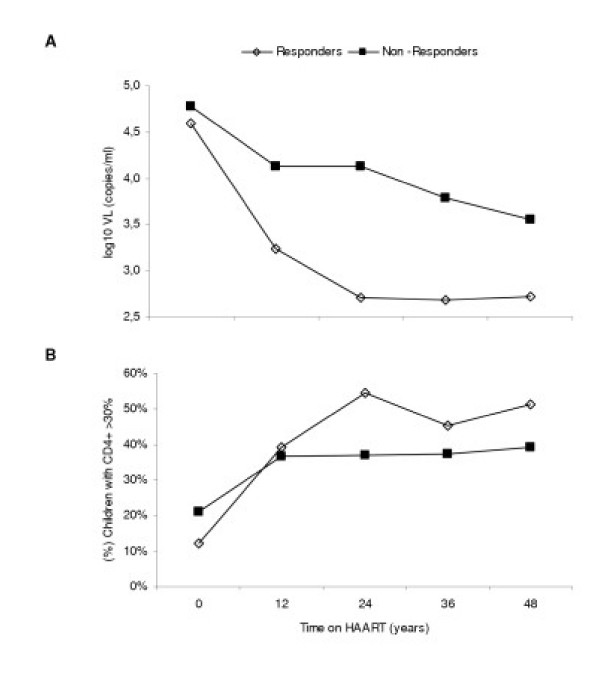
Evolution of mean plasma log_10 _VL (A) and percentage of HIV-infected children with CD4^+ ^>30% (B) grouped by long-term VL suppression (*Responders vs. Non-Responders*). ^a^n: number of children in the *Responders group*. ^b^n: number of children in the *Non-Responders group*. Differences between groups (*: p < 0.01).

Table 2 shows the first line HAART schemes. We did not find significant differences in PI used in first line HAART between groups. Moreover, there were 3320 VL assays performed on samples obtained from HIV-infected children during the 4 years of follow-up; 1054 (47.3%) of those did not reach the lower limit of detection of 400 HIV-RNA copies/mL. The VL tests performed were similar for the two groups, but the Responders group had a higher number of tests with VL ≤400 copies/ml. Moreover, the median of time between the first and last VL ≤400 copies/ml measurement was lower in the Responder group than the Non-responder group (Table 2). The Responders group had uVL after the 1^st ^year on HAART and the Non-Responders group had a low decrease of VL during the 4 years of follow-up (Figure 1A). Furthermore, the Responders group had a higher percentage of children with CD4^+ ^>30% than the Non-Responders group after 1^st ^year on HAART (Figure 1B).

**Table 2:** Characteristics of antiretroviral treatment and virologic parameters of vertically HIV-1-infected children during follow-up.

	***Responders***	***Non-Responders***
**HAART during follow-up**		
NNRTI or PI in first-line of HAART ^(b)^		
Ritonavir	4 (12.1%)	32 (25.1%)
Saquinavir	6 (18.2%)	12 (9.4%)
Indinavir	8 (24.2%)	28 (22.1%)
Nelfinavir	10 (30.3%)	44 (34.6%)
Amprenavir	3 (9.1%)	5 (3.9%)
Nevirapina	0 (0%)	6 (4.7%)
Efavirenz	6 (18.1%)	10 (7.8%)
ART-protocols switches ^(a)^	1 (0; 4)	1 (0; 11) *
Drugs switches ^(a)^	1 (0;7)	3 (0;12) *
Adherence >95% ^(b)^	30 (90.9%)	61 (48%) *
**VL tests**		
Total VL tests ^(a)^	20 (6; 34)	21 (3; 48)
Number of tests with VL ≤400 copies/mL ^(a)^	15 (5; 28)	3 (0; 23) *
Time to first uVL measure ^(a)^	3.7 (0.2; 12)	29.4 (0.9; 59.8) *
Time to last uVL measure ^(a)^	49.8 (16.1; 59.6)	57.5 (31.8; 60) *

Values are expressed as: a) median (min; max), and b) absolute (percentage). ART: antiretroviral therapy; VL: viral load; PI: protease inhibitor; NNRTI. Differences between groups (*: p < 0.01).

Table 3 shows the mean of %CD4^+ ^and percentage of HIV-infected children with CD4^+ ^≥25% during the 4 years of follow-up stratified by virological response to HAART. We observed an initial and significant fast increase in CD4^+ ^counts from baseline to 12 months on HAART in both groups (p < 0.01). Interestingly, there were children on HAART with sustained CD4+ increases but with detectable VL. *Non-Responders group *was sustained CD4+ increases and most of theses HIV-infected children maintained high CD4^+ ^level counts (≥25%). The *Non-Responders group *also reached a plateau between 26% and 27% CD4^+ ^at 12 months of follow-up that remained stable during the following 3 years (Table 3). However, the *Responders group *reached a plateau between 30% and 32% CD4^+ ^at 24, 36 and 48 months of follow-up. We found that the *Responders group *had higher CD4^+ ^count values and a higher percentage of children with CD4^+ ^≥25% than the *Non-Responders group *(p < 0.05) after month 12 (Table 3).

**Table 3:** Summary of %CD4^+ ^T-cell and percentages of HIV-infected children having CD4^+ ^≥25% at 0, 12, 24, 36, and 48 months of follow-up stratified by long-term virological response to HAART. The groups were compared by General Lineal Model and Logistic Regression analysis adjusted.

	**CD4^+ ^T-cells (%)**					**Percentage of children with CD4^+ ^≥25%**			
		
**Follow-up**	**n.**	***Responders***	**n.**	***Non-Responders***	***p***	***Responders***	***Non-Responders***	**OR (CI95%)**	***p***
0 months	33	16.4 ± 1.9	123	18.7 ± 1.2	*0.190*	24.2%	31.4%	0.6 (0.2; 1.6)	*0.372*
12 months	31	27.5 ± 1.7	121	26.2 ± 0.2	*0.145*	64.5%	53.7%	2.6 (0.9; 7.3)	*0.071*
24 months	31	29.5 ± 1.3	115	27.2 ± 0.9	***0.010***	70.9%	57.4%	3.1 (1.1; 8.6)	***0.031***
36 months	30	31 ± 1.2	109	27.7 ± 0.9	***0.005***	83.3%	62.3%	4.4 (1.4; 13.6)	***0.009***
48 months	28	32.1 ± 1.3	97	27.9 ± 1	***0.004***	85.7%	62.8%	5.4 (1.5; 18.9)	***0.008***

OR: odd ratio; CI95%: confidence interval of 95%; p: level of significance.

## Discussion

The introduction of HAART represented a major breakthrough in the therapeutics of HIV-infected patients. However, the overall effectiveness of long-term HAART on HIV-infected children has been scarcely studied. Thus, to date, few studies reflect the evolution of a large cohort of HIV-infected children throughout long-term HAART [6]. To analyze this, we recruited a large group of HIV-infected children starting HAART and tracked their progress for 4 years. Our retrospective longitudinal study provides evidence from current clinical practice that HAART always show long-term sustained uVL only in a low proportion of children (20.6%) just as it has previously been showing in published results in adults [7].

CD4^+ ^recovery despite virological failure has been referred to as discordant responses and this phenomenon has been observed in children [8]. However, in children on HAART, levels of CD4^+ ^recovery were not comparable for virological responders and non-responders, indicating that CD4^+ ^recovery during virological suppression is more profound than during virological failure [8]. This may reflect the inhibitory effect of HIV on thymic function since a marked decrease or suppression in VL is necessary to allow the thymus to replenish the CD4^+ ^T-cells. Moreover, CD4^+ ^T-cells are productively infected by HIV, undergoing apoptosis induced by abnormal cellular activation when the VL is not controlled [9].

In children with virological failure and good immunological and clinical outcomes observations have that virological failure does not always equal clinical failure [10,11]. Others authors have suggested that in children VL suppression may not be the best way to clinically evaluate ART success as it seem to be for HIV-infected adults [7]. Addition, HIV-infected children on HAART usually have higher VL and lower virological response rates than adults [12]. Thus, maintaining constant or improving CD4^+ ^counts may represent alternative indices of ART success in children [13]. We found an increase of CD4^+ ^counts in the *Responders *and the *Non-Responders groups*. This indicates that VL suppression was not indispensable to the recovery of the immune system in vertically HIV-infected children. Moreover, only one *Non-Responders *child died during follow-up and another evolved to AIDS. We carried out indirect measurement between responders and non-responders (hospitalization rates, weight/height growth curves, and numbers of opportunistic infections), and we found an improvement in these indices but not find differences between groups (*data not shown*). Therefore, virological failure does not always equal clinical failure and virological suppression does not necessarily mean proper function. However, long-term suppression of VL allowed higher values of CD4^+ ^counts.

High VL is associated with immune system activation which is used as a predictive marker of virologic failure [14,15]. Moreover, viral suppression is a powerful predictor of CD4^+ ^increase [16]. About 30% of HIV-infected adults receiving HAART exhibit a sustained CD4^+ ^increase despite therapy failure, or they have persistently low CD4^+ ^counts despite a significant decrease in VL [17]. In this study, approximately 30% of HIV-infected children also had an increase of CD4^+ ^to a level ≥25% at 24 (57.39%-35.52%), 36 (62.38%-35.52%), and 48 (62.88%-35.52%) months.

The capacity for CD4^+ ^regeneration during long-term HAART has not been well defined. In our study, children reached a plateau in CD4^+ ^cell after 2 years on HAART just as others authors have published results in adults [18,19]. Moreover, Hunt et al. present strong evidence that CD4^+ ^counts continue to increase up to 4 years after HAART [20]. However, the rate of CD4^+ ^recovery in adults is slow and a steady state is not usually reached after 4 years most likely because the adult thymus is less functional than in children [21].

A limitation of our study was that we did not have virologic resistance data previous to HAART and during follow-up. Increasingly many clinicians and investigators are describing multi-drug resistant HIV among children who are extensively HAART experienced [22]. Moreover, high baseline VL and substantial but imperfect levels of adherence were associated with HIV-resistance [23] and may facilitate an earlier virological failure [22]. Therefore, adherence to medication is extremely important and may have a significant, effect unaccounted for in the interpretation of our results. All efforts were made by health personnel to improve adherence in each child. Traditionally, children's adherence to ART has been limited, even though response to therapy has been shown to be highly dependent on the patient's adherence [24,25]. In this study, the adherence to ART was not strictly monitored, as opposed to standard clinical trials. We also found that adherence to ART was the most important variable associated with long-term VL suppression (*data not shown*). However, therapy adherence is almost impossible to reliably measure, and accurately reproduce in a long-term dynamic cohort [7]. This low adherence in the *Non-Responders *group is not related to the level of training received by the health personal who provided the medical care. Furthermore, the number of doctor visits was similar in both groups of children, but the *Responders *group had a higher number of tests with VL ≤400 copies/mL and a lower time with the first uVL measurement than the *Non-Responders *group.

## Conclusion

In conclusion, long-term VL suppression induces large beneficial effects in immunological responses. However, it is not indispensable to recover CD4^+ ^counts (levels).

## Competing interests

The author(s) declare that they have no competing interests.

## Authors' contributions

- Salvador Resino had primary responsibility for protocol development, patient screening, enrollment, outcome assessment, preliminary data analysis, and contributed to the writing of the manuscript.

- Rosa Resino had primary responsibility for the collecting and recording data, and contributed to the writing of the manuscript.

- José María Bellón participated in analytic framework for the study, and contributed to the writing of the manuscript.

- Pediatricians: Juan Antonio León, Pablo Matín Fontelos, José Tomás Ramos, M^a ^Dolores Gurbindo Gutiérrez, M^a ^Isabel de José, and Luis Ciria were responsible for patient screening, and contributed to the writing of the manuscript.

- M^a ^Angeles Muñoz-Fernández supervised the design and execution of the study, the final data analyses, and the writing of the manuscript.

## Pre-publication history

The pre-publication history for this paper can be accessed here:


